# Unconventional Germination in Terrestrial Plants: A Counterintuitive Case in Desiccation-Sensitive *Garcinia aristata* (Clusiaceae) Seeds Showing Seedling Growth Without Roots

**DOI:** 10.3390/plants13233269

**Published:** 2024-11-21

**Authors:** Ganesh K. Jaganathan, Jorge A. Sánchez, Mayté Pernús, Baolin Liu

**Affiliations:** 1 Germplasm Conservation Laboratory, Department of Health Science and Engineering, University of Shanghai for Science and Technology, Shanghai 200093, China; blliuk@163.com; 2 Institute of Ecology and Systematics, Ministry of Science, Technology and Environment, La Habana 200093, Cuba

**Keywords:** adventitious roots, desiccation, embryo, inverse dormancy, recalcitrant

## Abstract

Unconventional germination, wherein shoots emerge and establish true leaves before the root emerges, is only found in Zosteraceae. In *Garcinia*, germination proceeds with the primary root emerging, followed by shoot emergence on the opposite side, but before leaf differentiation, adventitious roots emerge at the base of the shoots. However, germination and survival mechanisms in several *Garcinia* species are still unclear. We subjected freshly collected *G. aristata* seeds to an imbibition test, and germination was evaluated at various temperatures and light conditions. Desiccation sensitivity assessments were made at different stages of drying. The effect of natural drying (ambient storage) on germination was assessed by leaving the seeds outside in natural conditions. Seeds of *G. aristata* with a moisture content (MC) of 67% had more than 95% germination only at 25 and 25/30 °C both in light/dark and darkness, but at 25/40 °C only 10% germinated. In 4% of the seeds, germination incepted with primary shoot emergence, and a secondary (adventitious) root emerged just before leaf differentiation. More than 95% of the seeds germinated with only a secondary root and shoot emerging concurrently. Drying fresh seeds above silica gel to 30% MC resulted in complete viability loss. Seeds stored at ambient conditions germinated without external water, and had no primary or secondary root, and the emerging shoot continued to grow into seedlings. A root develops in these seeds only when water becomes available. *G. aristata* seeds are desiccation-sensitive and non-dormant. When no external water is available, *G. aristata* seeds can germinate with shoots and establish seedlings. This is the first report on germination and successful seedling establishment without roots in *Garcinia*.

## 1. Introduction

Germination is the most critical event in the life history of spermatophytes [1,2]. Invariably, germination leads to radicle protrusion from the external covering layers of seeds or dispersal/germinating unit [hereafter germinating unit or seeds] and establishes a root system, followed by the development of a shoot system and “eventually” leaves [3,4]. Indeed, this pattern occurs in most contemporary species; consequently, radicle extension by 2 mm outside the germinating unit has been recommended as a criterion to assess germination by various regional and international authoritative bodies, including the International Seed Testing Association [5]. However, germination is much more complex than this simplistic overview in at least *c.*10–20% of global species, where the first structure emerging from the germinating unit is not a radicle per se but a range of other structures, including the hypocotyl, coleorhiza, coleoptile, etc. Baskin and Baskin [6] recently drew attention to such germination behavior, as they do not fit the ‘radicle emergence’ criterion, and stressed that more caution is needed in studies involving those species.

Regardless of the emerging structures from the germinating unit, a root system is permanently established before onsetting a shoot system. The only exception to this otherwise ubiquitous rule is a few species from Zosteraceae. As early as 1902, Gibbs demonstrated that *Phyllospadix* seeds had their hypocotyl and plumule emerge first, and they established several leaves 6 months before roots emerged. Tutin [7] and Taylor [8] found identical results in *Zostera marinia*, demonstrating that roots are not necessary for the leaves to form and function independently. These studies underpinned that leaf emergence followed by root emergence could be much more common in Zosteraceae. Perhaps the most detailed assessment of this subject was presented by Kuo et al. [9], who clearly showed in *Phyllospadix iwatensis* that the seeds have dormancy and germination onsets, with leaves emerging first and root emergence being delayed for six months after the first leaves emerged. Thus, if the seeds have dormancy, there is a delay between dispersal and germination, but leaves emerge first, and radicle emergence in the germination process is further delayed. Subsequent studies have shown similar germination patterns in *Zostera marinia* maturing in other locations [10]. Similarly, evidence is emerging to show this germination pattern in a wide range of Zosteraceae species, e.g., *P. japonicus* [11].

A more unusual germination pattern occurs in the genus *Garcinia*, belonging to Clusiaceae. While the radicle develops into the primary root system, the hypocotyl plays a crucial role in seedling establishment by lifting the cotyledons, facilitating root–shoot transition, and regulating growth in response to environmental cues, without directly developing into roots itself. Unlike other families, *Garcinia* species have the primary root and the shoot appearing on opposite sides, but subsequently, just before leaf differentiation, an adventitious root develops at the base of the shoot, which matures into the main root, often referred to as *Garcinia*-type germination [12,13,14,15,16,17,18,19,20]. However, Malik et al. [20] showed that the shoot and primary and secondary roots emerged on the same side in *G. indica*, *G. cambogia*, and *G. xanthochymus*. This germination pattern has been previously described in other members of Clusiaceae, e.g., *Symphonia globulifera* [21] and *G. kola* [22]. Further, *Garcinia* seeds of many species can “have multiple embryos” or “germinate with multiple seedlings”. For instance, when the fully intact seed is cut into pieces artificially with a scalpel or naturally by animals, each piece could produce a seedling [18,20,23]. However, the prevalence of *Garcinia*-type germination across the c. 300 known *Garcinia* species remains uncertain, as research has primarily focused on a limited number of species, mainly found in Southeast Asia [24].

The moisture content of the seeds at the time of dispersal influences germination. Desiccation-tolerant or orthodox seeds [sensu [25]] require water imbibition to onset germination. Desiccation studies conducted on a handful of *Garcinia* species have shown that the moisture content at the time of dispersal is high, typically above 35% on a fresh weight basis (fwb), and drying to a moisture range of approximately 25% fwb results in complete mortality [26,27,28,29,30,31,32,33], thus indicating that these seeds are desiccation-sensitive, i.e., recalcitrant [sensu [25]]. Because dormancy in desiccation-sensitive seeds is rare yet not mutually exclusive [34,35], dormancy in *Garcinia* species has received scant attention. The results of these dormancy studies are equivocal, with some studies showing that *Garcinia* species produce non-dormant seeds, e.g., *G. lucida* [36], but others indicating that seed germination is delayed when sown in appropriate conditions [29,30,37,38,39,40], presumably indicating the presence of physiological dormancy (PD), wherein the embryo lacks the potential to break open the seed coat. Baskin and Baskin [6] included *Garcinia* under a new subclass of PD called ‘Hypocotylar’ and suggested that warm temperatures are required to break PD and the shoot is produced after the root is produced. Nonetheless, Viana et al. [41] claimed that seeds of *G. gardneriana* have recalcitrant seeds with physical dormancy (PY), which results from the water-impermeable nature of the seed/fruit coat. They further stated that this paradoxical combination could arise due to environmental pressure. When coupled with other inferences in previous studies suggesting epicotyl dormancy, where the root emerges as soon as seed dispersal occurs but shoot emergence is delayed, e.g., *G. kola* [14], dormancy in *Garcinia* species requires further investigation.

To date, no other species outside Zosteraceae has been known to have leaves emerging before root emergence. Here, we describe this germination pattern in Clusiaceae. *Garcinia aristata* (Griseb.) Borhidi (≡*Rheedia aristata* Griseb.) is a slow-growing evergreen tropical tree that grows up to 15 m [42]. The species is endemic to the Caribbean and naturally distributed in Cuba and the Dominican Republic [43]. In Cuba, *G. aristata* is a critically endangered species to the extent it is among the top 60 species protected by the Cuban forest system. Despite such conservation actions, overexploitation for medicinal and timber use, habitat loss, degradation caused by deforestation, agricultural activity, livestock, invasive exotic plants, and climate change put more pressure on natural regeneration [44,45]. This fate is shared by 11 endemic Cuban species of *Garcinia* [46].

The standing populations of *G. aristata* have been observed only in mesophyllous and microphyllous evergreen forests, where water is readily available throughout summer [46]. Moreover, climate change is seriously affecting the germination phenology of numerous species across the globe and Caribbean regions, and our knowledge of how a changing climate might affect seeds in general and, particularly, recalcitrant seeds, such as *Garcinia*, is rudimentary. Indeed, there is evidence of hampered germination in Cuban tree species that colonize tropical evergreen forests [47,48].

In this work, we studied the germination and seedling growth of *G. aristata* to gain more understanding of the following questions: (1) Do seeds have dormancy and, if so, what is the class of dormancy? (2) Does germination occur only at specific temperatures and light conditions? (3) What are the germination processes of the seeds? (4) Are seeds sensitive to desiccation? (5) How do low water potential and ambient storage affect seed germination?

## 2. Materials and Methods

### 2.1. Seed Collection and Habitat Characteristics

Fully ripened yellow color fruits of *G. aristata* were collected on 24 July 2017, in the National Botanical Garden in the Boyeros municipality of Havana, Cuba (22°99′34″ N, 82°33′49″ W) from seven trees with the help of a telescopic rod. The change in the color of the fruit from green to yellow indicated that the seeds had reached maturity, and our collection time was synchronized with the natural dispersal time. The collected fruits were pooled together and washed under running tap water before extracting the seeds by crushing them. Typically, a fruit contained one seed, but rarely two or three. A scalpel was used to scrap out any fruit remains. Subsequently, the clean seeds were sterilized in 1% sodium hypochlorite for 5 min, washed thrice with distilled water to avoid fungal infections, and then placed on filter paper in iron trays at ambient laboratory conditions (25 ± 2 °C; 60% RH) for 48 h. Seeds were randomly sampled for experiments, which began immediately. However, seeds used to assess general characteristics, for the imbibition test, and to assess ambient storage were fresh, unsterilized seeds.

The climate of the seed collection site is subtropical and seasonally humid, with a rainy season that extends from May to October (summer season) and a dry period from November to April, the latter corresponding to the lowest temperatures for Cuba [49]. The mean annual rainfall is 1541 mm, and the mean annual temperature is 24.3 °C, with an average temperature of 31.8 °C during the warmest month and a lowest average monthly temperature of 26.7 °C, according to the WorldClim 2 [50].

### 2.2. Seed Characteristics

Seed dimensions (length, width, and depth), shape, fresh and dry mass, moisture content (on a fresh weight basis), and coat and embryo weight were determined from a random sample of 100 seeds. Seed length, width, and depth measurements were made using a Mitutoyo Caliper with a precision of 0.02 mm. The seed shape index (variance of seed dimensions) was calculated as described by Thompson et al. [51]. Before calculating the variance, each value of the seminal dimension was divided by the length value. Thus, a spherical seed will present a variance value of 0, while for an elongated or flattened one, its variance can be up to 0.33. This is because in a perfectly spherical seed, the L = W = H, resulting in ratios of 1,1, and 1; thus, the variance of these identical values would be 0. In contrast, larger variance values indicate seeds are more elongated or flattened compared to a sphere. The fresh mass was determined by individually placing the seeds on a scale (Sartorius, with 10^−4^ g accuracy), and the seed coat ratio (seed coat dry mass/whole seed dry mass) (SCR) was determined following the methods described by Daws et al. [52]. The probability index of sensitivity to desiccation (*P*) was also determined based on biometric data of the seeds (total dry mass and seed coat ratio), according to Daws et al. [52]. Briefly, this method takes into account the seed mass and SCR to predict desiccation-sensitivity. Thus, as the seed mass increases, the probability of desiccation sensitivity generally increases. On the other hand, the probability of desiccation sensitivity decreases with an increasing SCR. The model considers a seed to be desiccation-sensitive when P(D-S) > 0.5, and desiccation-tolerant when P(D-S) < 0.5. The moisture content was calculated by comparing the fresh mass after drying the seeds at 103 ± 2 °C for 17 h [5].

### 2.3. Seed Imbibition

The ability of seeds to absorb water (or not) was determined in an imbibition test, using seven replicates of 20 seeds placed on two layers of filter paper moistened with distilled water in 12 cm diameter Petri dishes incubated at 25 ± 2 °C under diffused white light. After 48 h of imbibition, seeds of each replicate were removed separately, placed in a soft tissue pad, patted dried to remove surface water, and then weighed on a balance (0.00 g). The percentage increase in mass (PIM) was calculated following Baskin et al. [53]: PIM = [(M2 − M1)/M1] × 100, where M1 and M2 represent the initial fresh mass of the seed (zero time) and the mass of the seed after 48 h on a moistened substrate, respectively.

### 2.4. Effect of Temperature and Light on Seed Germination of Fresh Seeds

To determine the effect of temperature and light, seven replicates of 20 seeds were germinated in 12 cm diameter Petri dishes containing a double layer of filter paper moistened with distilled water placed in growth chambers (Medcenter Einrichtungen GmbH, FRIOCEL 111 L, Munich, 2.8 Germany) at a constant temperature of 25 °C and alternating temperatures of 25/30 °C, 25/35 °C, and 25/40 °C either in dark or dark/light. The light had an illumination of 40 μmol m^−2^s^−1^; 400–700 nm of cool-white fluorescent light was provided for 12 h at 25 °C in seeds experiencing constant temperature or during the warm phase of the cycle. The Petri dishes of seeds in the dark were wrapped with a double layer of aluminum foil to preclude light from reaching the seeds. The tested temperature range mirrored the soil temperature variations observed in humid forests of western Cuba during the month when the seeds were collected. These temperatures represented conditions from the forest interior to large open clearings [47,48]. The temperature range of 25/40 °C was used to investigate the influence of climate change on germination.

### 2.5. Effects of Drought Stress on Seed Germination

The effect of drought stress (i.e., low water potential) on germination was determined by incubating seeds at 0, −0.3, −0.6, −0.9, and −1.2 MPa. Different water potentials were achieved by diluting 0, 151.40, 223.66, 279.29, and 326.26 g/L of polyethylene glycol-6000 (PEG 6000, Merck Group, Darmstadt, Germany), respectively, in water at 25 °C, as described by Villela et al. [54], based on Michel and Kaufmann [55]. Seeds were then set to germinate on a double layer of filter paper soaked in at least 12 mL of the corresponding PEG solution in 12 cm Petri dishes, sealed with plastic film to prevent evaporation of the solution. Seven replicates of 20 seeds were used for each treatment and placed in an incubator at 25 °C with 12 h light conditions. Germination was evaluated daily over 30 days. Non-germinated seeds were rinsed with distilled water and transferred to 12 cm diameter Petri dishes containing a double layer of moistened filter paper. These seeds were germinated at 25 °C with 12 h light for 25 days. Seeds that remained ungerminated throughout were subjected to a cut test to evaluate the internal structures.

### 2.6. Desiccation-Sensitivity Assessment

The germination capacity of freshly collected seeds at different moisture levels was determined by desiccating four replicates of 45 seeds in air-tight glass vessels (3 L capacity) containing activated silica gel. The seeds were desiccated for 1, 2, 3, 4, and 5 weeks under laboratory conditions (25 ± 2 °C; 60–65% RH). At the end of each drying period, germination assessments were carried out by incubating four replicates of 25 seeds on Petri dishes containing double-layered moist filter paper at 25/30 °C (12 h light conditions). The remaining four replicates of 20 seeds were used to determine the moisture content, as described above. Germination tests were terminated after four weeks. The final germination percentage and first germination time (in each replicate) were evaluated.

### 2.7. Effects of ‘Ambient Storage’ on Seed Germination

To determine how quickly the seeds can dry under ambient conditions and whether this would influence germination, 362 fresh seeds were placed on two layers of dry filter paper in an iron tray (44: length × 34: width × 4: thickness, cm). These trays were incubated at room temperature with 12 h of light provided using 400–700 nm of 30 μmol m^−2^s^−1^ cool-white fluorescent lamps. The physical change in the seeds and any germination were monitored for three months. The temperature and relative humidity of the laboratory during storage were recorded using a HOBO U12 Data Logger, for which a thermocouple was placed inside the tray that contained the seeds. The intact and ungerminated seeds were subjected to a cut test to evaluate the internal structures. We refer to this group of seeds as ‘ambient storage’.

### 2.8. Documenting the Morphological Progression in Germination of Fresh and ‘Ambient Storage’ Seeds

Preliminary germination tests showed that the shoot (i.e., the epicotyl) was the first to emerge; therefore, a seed was defined as germinated when the shoot emerged ≥ 2 mm. However, we documented the emergence of primary and secondary roots, from which side they emerged, and their growth. Visible changes were observed and documented daily for 30 days, except for seeds held in complete darkness, for which morphological changes were documented after 30 days. The seeds that did not germinate in light and dark at 25/40 °C were transferred to 25/30 °C with 12 h of light, and morphological changes in seeds were monitored for an additional 20 days.

### 2.9. Statistical Analysis

Descriptive analyses (e.g., mean, standard deviation, and coefficient of variation) were used to describe the seed traits. The final germination percentage was calculated as the mean of the seven replicates and expressed with standard errors. A generalized linear model (GLM) was used to evaluate the effect of temperature, light, and their interactions on seed germination, assuming a binomial error distribution and using the Logit Link function. The model with the best fit was selected using Akaike’s information criterion and the Bayesian information criterion [56]. A generalized linear model was used to evaluate the effects of drying periods on final germination percentage, but the time to start germination was analyzed assuming a Gaussian distribution and using the identity link function. Data were analyzed using the InfoStat program, which implements a user-friendly interface for the platform R. Means were compared with a post hoc DGC test.

## 3. Results

### 3.1. Seed Characteristics and Imbibition

Fully matured fruits of *G. aristana* were yellow; each contained one large, heavy, and bean-shaped seed with an average mass of 2.01 ± 0.68 g. The mature seeds were enclosed in a dark brown seed coat and lack endosperm. Inside, the embryo, ranging from yellow to light green in color, consisted primarily of a large hypocotyl–radicle axis that occupied the entire seed cavity. The embryo was approximately 6-fold heavier than the coat, and the seed coat embryo ratio was 0.11 ± 0.04 (Table 1). The probability of desiccation sensitivity value was 0.82 ± 0.13 (Table 1). All the morphophysiological traits studied showed considerable variability, except for the initial moisture content and the increase in seed mass after imbibition (Table 1). The average moisture content of the fresh seeds was 67.22 ± 3.38% on a fresh weight basis, with a maximum value of 76.10 and a minimum value of 61.04% (Table 1). When incubated in a wet substratum, the mass of the seeds increased by 12.02 ± 2.21% within 48 h (Table 1).

### 3.2. Effect of Temperature and Light on Seed Germination on Fresh Seeds

**Figure 1:**
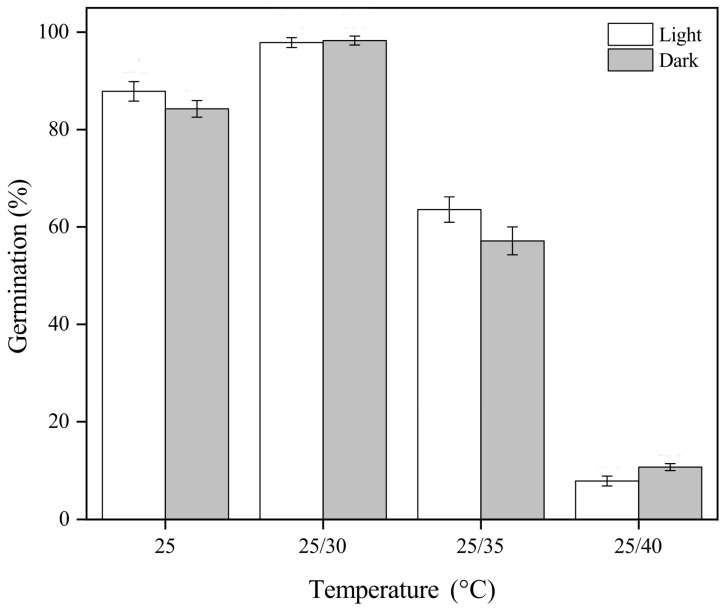
Final germination percentages (mean ± SE of *Garcinia aristata* seeds incubated under different temperatures and light conditions. Temperature significantly affected germination, while light exposure had no statistically significant impact.

**Figure 2:**
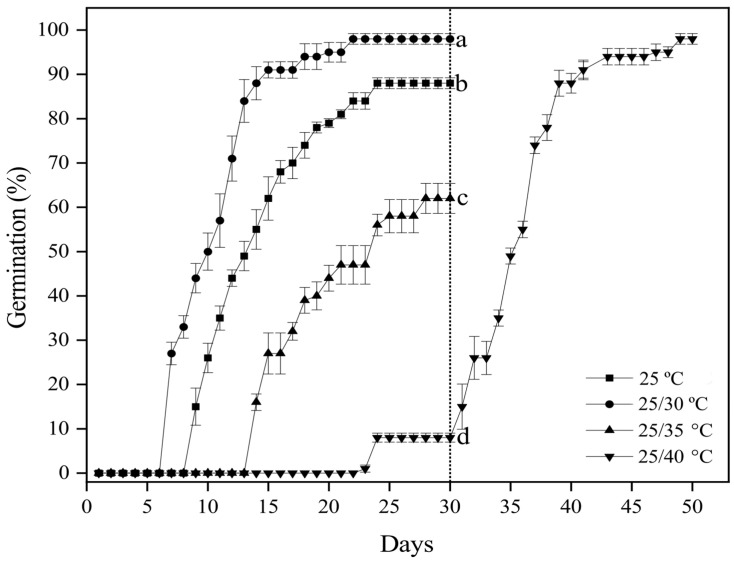
Cumulative germination percentages (mean ± SE of fresh *Garcinia aristata* seeds incubated at different temperatures. Different letters indicate significant differences after 30 days when germinated at four different temperatures (*p* ≤ 0.05). The germination percentage of seeds incubated at 25/40 °C after 30 days (shown with a dotted line) was determined after moving the seeds to 25/30 °C.

The final germination percentage of fresh *G. aristata* seeds was significantly affected by temperature (*p* < 0.001; Table 2; Figure 1) but not by light (*p* > 0.05; Table 2) or the interaction between temperature and light (*p* > 0.05; Table 2; Figure 1). At 25 °C, final germination reached above 80% in light and darkness. However, regardless of light, seeds incubated at 25/30 °C had 98% germination, but those at 25/35 and 25/40 °C only had around 50 and 10% germination, respectively (Figure 1). Nonetheless, when the non-germinated seeds were moved from 25/40 °C to 25/30 °C, the germination percentage significantly increased (Figure 2). The final germination percentage of seeds incubated at 25/40 °C for 30 days and then moved to 25/30 °C for an additional 20 days was 98 ± 1.22%, identical to the seeds incubated at 25/30 °C (Figure 2). For seeds incubated at 25/30 °C, germination began after seven days and reached 98% within 22 days, whereas at 25 °C, germination began after 9 days, reaching only 87% germination after 30 days (Figure 2). On the other hand, for the seeds incubated at 25/40 °C, it took 22 days for germination to start and they had only 10% germination after 30 days. However, within 20 days after moving the seeds from 25/40 °C to 25/30 °C, germination increased to 98% (Figure 2).

### 3.3. Desiccation Sensitivity Assessment

**Figure 3:**
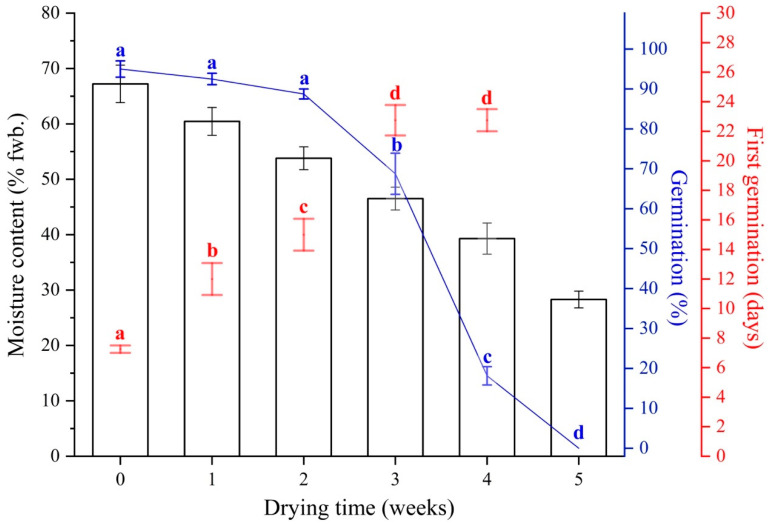
The relationship between moisture content, germination percentage (blue line with error bars), and time taken for first germination (just the error bars) after drying fresh *Garcinia aristata* seeds for different durations above silica gel. Different lower-case blue letters indicate significant differences between groups for germination. Different lower-case red letters indicate significant differences between groups for mean germination time. Error bars represent standard errors.

Before drying, the freshly collected seeds with 67% moisture content had 95% germination at 25/30 °C. All seeds germinated in 20 days, most between 7 and 10 days (Figure 2 and Figure 3). Silica gel drying was slow, and there was no apparent loss in moisture content or germination when tested at the end of the first week (Figure 3). There was a slight decrease (non-significant) in germination percentage after two weeks of drying, but moisture content remained above 50%. However, there was a sharp decline in germination percentage after continuously drying the seeds over 2 weeks, with only 68 and 18% germinating when the moisture content reached 46 and 39% after 3 and 4 weeks, respectively (Figure 3). This significant drop in germination percentage continued, and no germination was observed after drying the seeds for 5 weeks, at which stage the moisture content remained at 28%. The first two weeks of drying did not influence the mean germination time; despite a significant difference in moisture content, germination was completed within 25 days. However, the germination percentage differed significantly between 3- and 4-week-dried seeds; the first germination time did not show such a difference (Figure 3).

### 3.4. Effects of Drought Stress on Seed Germination

None of the freshly matured seeds tested at any lower water potentials were able to germinate after 30 days. Subsequent movement of seeds to filter paper containing water at various water potential levels showed no signs of germination. However, the cut test indicated that the internal structures of the seeds were unaffected.

### 3.5. Effects of ‘Ambient Storage’ on Seed Germination

**Figure 4:**
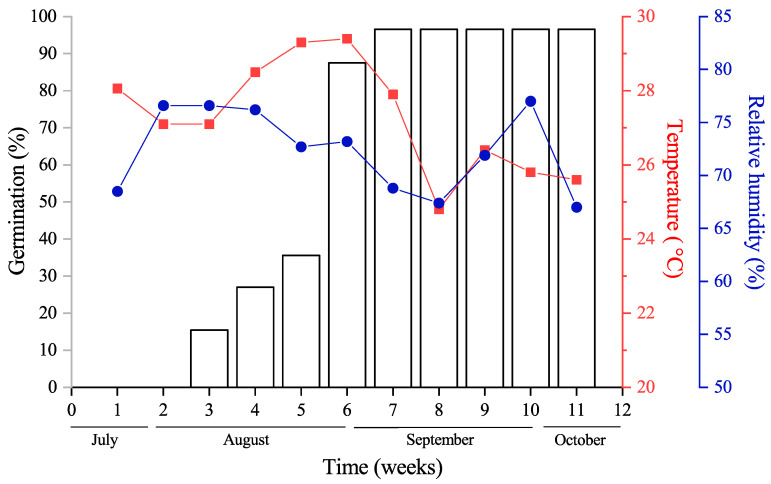
The mean temperature and relative humidity experienced during ‘ambient storage’ of *Garcinia aristata* seeds. The bars indicate the weekly cumulative germination percentage.

**Figure 5:**
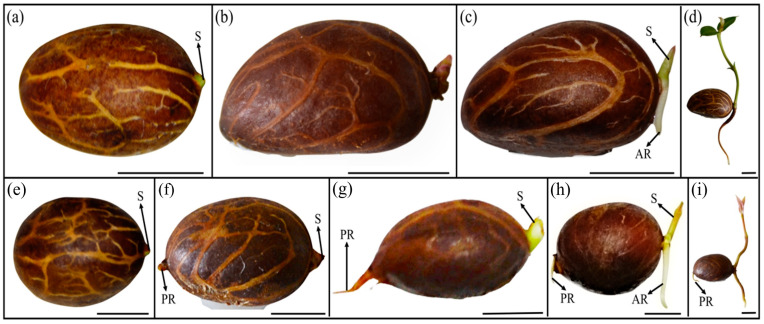
Two different forms of germination observed in fresh *Garcinia aristata* seeds with and without primary root. Germination of *Garcinia aristata* seeds without primary roots (**a**) 2 days, (**b**) 7 days, (**c**) 15 days, and (**d**) 25 days after germination. Germination of *Garcinia aristata* seeds with primary and secondary roots: (**e**) 2 days, (**f**) 7 days, (**g**) 15 days, (**h**) 21 days, and (**i**) 28 days after germination. S, shoot; AR, adventitious or secondary root; PR, primary root. Scale = 0.5 cm.

After 21 days of ‘ambient storage’ with no external water supplied, shoot emergence (i.e., germination) was observed in 56 seeds (15.46%) of 362 seeds (Figure 4), and after 28, 35, 42, and 49 days, 27.0, 35.63, 87.56, and 96.68% of seeds germinated, respectively. A more significant increase in germination was obtained at 42 days of storage (52.0%). At the end of three months, 12 seeds (3.31%) did not germinate, and a cut test revealed these seeds were dead from drying; the emerged shoot reached the true leaf stage with an average height (mean ± SD) of 5.3 ± 0.09 cm (Figure 4 and Figure 5). These seedlings had neither primary nor secondary adventitious roots (see below). However, once in contact with water, these seedlings had secondary root emergence within two or three days, regardless of the seedling stage. The average temperature experienced by seeds during ‘ambient storage’ for three months was 24.7 °C (maximum: 26.1 °C and minimum: 24.3 °C), and the relative humidity was 65% (maximum: 71.5% and minimum: 54%).

### 3.6. Germination and Seedling Structures of Fresh and ‘Ambient Stored’ Seeds

**Figure 6:**
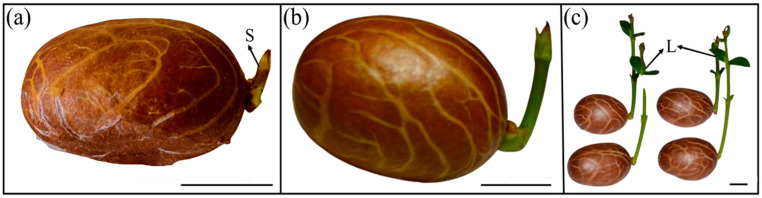
Germination and seedling growth of ‘ambient-stored’ *Garcinia aristata* seeds with shoots emerging without roots after (**a**) 14 days, (**b**) 45 days, and (**c**) 90 days. The seedlings established fully functional leaves without roots. S, shoot; L, leaves. Scale = 0.5 cm.

More than 95% of the *G. aristata* fresh seeds germinated with the emergence of the shoot, followed by the adventitious root emerging from the base of the shoot just before the leaf differentiated (Figure 5a–c). The primary root never emerged in this group of seeds (Figure 5d). In another group of seeds, the shoot emerged first (Figure 5e), and within three to four days, the primary root emerged on opposite poles (Figure 5f,g). Immediately after the emergence of the primary root, the secondary root emerged from the base of the shoot, and approximately 25 days after germination, true leaves appeared (Figure 5h,i). This type of germination only occurred in 4.28% of those sown. Both these groups had the shoot emerging first (Figure 5a,f). A third group of seeds had shoot emergence, but no primary or secondary root emerged (Figure 6a,b). The shoot continued growing and establishing true leaves without roots (Figure 6c). This pattern was only observed in ‘ambient storage’ seeds without an external water supply.

## 4. Discussion

The initial moisture content of 67% observed in the present study on *G. aristata* is on the higher end of the spectrum when compared with a range of species across *Garcinia* species, typically between 34.92% in *G. atroviridis* and 61.12% in *G. mangostana* [27]. The higher moisture content does not necessarily indicate immature unripe seeds because the extracted seeds are dark brown, but immature *Garcinia* seeds tend to be cream-white [31]. Despite these differences, our results agree with other traits found in *Garcinia* seeds. Firstly, all the species investigated hitherto, including *G. aristata* (Table 1), have no endosperm or cotyledons or any other storage tissues, and the embryo (nic axis) occupies most of the seeds [26,27,29,30,31,32]. Secondly, the seed size and shape are in the range reported previously [12,19].

Temperature is a crucial factor affecting *G. aristata* seed germination. A higher temperature above 30 °C seems to inhibit germination, which could be ubiquitous among *Garcinia* species. Consequently, studies using higher germination temperatures ascribed failed germination to dormancy. For instance, Liu et al. [37] showed that *G. cowa* did not germinate after 120 days when incubated at 30 °C. Yet, this assertion does not explain the more extended period required for germination at 25 °C [32]. A more common finding with dormancy in *Garcinia* species is that the seed coat imposes a barrier to germination; thus, removing the seed coat increases water uptake and the germination rate and percentage [29,30,57]. As such, this indicates that PD might be a possibility. However, our results showed that *G. aristata* seeds germinated within 30 days in light and darkness when incubated at 25 °C and 25/30 °C but not at 25/40 °C (Figure 1 and Figure 2). In addition, germination was hampered at lower water potentials, but viability was unaffected. Seeds experiencing a lower potential after dispersal could remain ungerminated. We surmise that the germination rate and percentage of *Garcinia* seeds would depend mainly on the temperature at which germination tests are carried out and the maturation state. In this regard, it must be stressed that a slower water uptake does not necessarily imply the seed coats are impermeable to water, i.e., PY. Thus, the PY claim in *G. gardneriana* by Viana et al. [41] and the other studies making such inferences stems from the fact that these authors equated the physical barrier of the seed coat impeding embryo protrusion with PY.

There is general agreement that seeds of *Garcinia* species do not survive desiccation, and our results strongly support this assertion (Figure 3). Indeed, the probability of desiccation sensitivity estimated based on Daws et al. [52] had a value of 0.82. From this, along with the desiccation results showing that seeds of *G. aristata* used in the present investigation lost more than half of their viability when dried to *c*. 35% MC (Figure 3), which is the critical moisture content (CMC), we conclude the seeds are highly desiccation-sensitive. As such, this result contrasts with the CMC of 20–30% reported in most—if not all—*Garcinia* species studied thus far [26,27,29,58]. Further, the time taken to reach the CMC when dried above silica gel was 3–4 weeks in the present study, but Normah et al. [27] reported that seeds of *G. mangostana*, *G. atrovirdis*, and *G. prainiana* took only 2–3 days to reach the CMC. Likewise, Malik et al. [26] showed that *G. indica*, *G. cambogia*, and *G. xanthochymus* seeds dried above silica gel lost complete viability within two days. Working with *G. kola*, Joshi et al. [29] also reported that fresh *G. kola* seeds with 43% MC took 3–5 days to reach the CMC when dried above silica gel.

Our germination and seedling growth assessment revealed that seedling establishment in *G. aristata* is much more complex, with three patterns, namely (1) the shoot emerged, and the primary root emerged on the opposite side, with adventitious roots developing at the base of the shoot before leaf differentiation (Figure 5g–i); (2) the shoot emerged, but the primary root never emerged, with only adventitious roots shortly emerging at the base of the shoot (Figure 5b–e); and (3) the shoot emerged and no primary or secondary root emerged (Figure 6a–c). However, shoot emergence without a root is found only in ‘ambient storage’ seeds. Because there was no delay in root emergence after shoot emergence, the occurrence of epicotyl physiological dormancy (ePD) can be ruled out, which agrees with results found on *G. kola* [59], *G. brasiliensis* [19], and *G. gummi-gutta* [12]. Normah et al. [27] showed that in *G. prainiana*, there was no emergence of the adventitious root, and the primary root grew into the main root. However, in their study, *G. mangostana*, *G. hombroniana*, and *G. atroviridis* followed typical *Garcinia*-type germination.

Of the three germination patterns observed, failure of root emergence occurred only during ‘ambient storage’, and germination of seeds in the presence of water always had either a primary or secondary root emergence. Intriguingly, seeds experiencing ‘ambient drying conditions’ continued to grow a shoot, and the adventitious roots emerged within days when water became available, irrespective of the seedling size. Dormancy could synchronize germination with the growing season, but we argue that the seeds of *G. aristata* are non-dormant, even though they closely resemble the ‘inverse epicotyl’ dormancy reported in Zosteraceae [6]. Dormancy is a temporary failure in seed germination even if seeds experience all the conditions, including water, oxygen, soil, etc. [1,2,60]. Because the seed coat is permeable to water, intact seeds germinated within 30 days, and there was fully functional shoot emergence and failure of root emergence only occurred in seeds not provided with external water, and so we rule out the possibility that the seeds are dormant. However, seedlings without roots could be an adaptive mechanism to cope with post-dispersal dry conditions. Desiccation-sensitive seeds do not necessarily need external water for germination, as the moisture content is high at dispersal and proceeds to embryo growth [61]. Nonetheless, the lack of germination in seeds exposed to salt-induced water potential solutions remains inexplicable, especially given the shoot emergence in open-air stored seeds with lower water potential. We hypothesize that direct contact between salt solutions and seeds creates a unique aqueous microenvironment, distinct from ambient storage conditions. Thus, the extent to which *G. aristata* seeds can continue to grow without roots remains to be explored in future studies.

The finding that *G. aristata* seeds can grow seedlings without roots is puzzling. Currently, it is not known if this mechanism is common in other *Garcinia* species because all the germination studies, thus far, have been conducted in a medium supplied with water. Similarly, the ecological significance of this mechanism is also unclear. It is plausible that the high moisture content of *G. aristata* seeds, and the requirement to maintain a sufficient moisture content above the CMC until germination, played a critical role in the evolution of this unusual germination. Unlike most *Garcinia* species, we found the initial MC and CMC are slightly higher for *G. aristata*. In addition, to the best of our knowledge, the slower drying rate reported in the present study with seeds requiring 3 weeks of drying to reach the CMC has never been reported, although variation in initial moisture content within species has been reported for seeds maturing at different locations. For instance, seeds of *G. kola* from Nigeria had a fresh MC of 50.8% [62], but those from Ghana had 58% [58]. A drop in moisture content may lead to the onset of stress response [63]. From the studies conducted on *G. kola*, seeds under stress could germinate with abnormal root systems [22]. We also found that *G. aristata* seeds experiencing lower water potentials did not germinate, although the viability seemed unaffected. Perhaps the requirement to maintain a higher moisture content during post-dispersal survival results in shoot emergence as a mechanism to avoid desiccation. The post-dispersal conditions from the seed collection site indicate a dry season between August and September, before the arrival of the rainy season. This response could have evolved to counteract the drying pressure. The effect of water potential on the germination of *Garcinia* requires further detailed study.

Similarly, shoot emergence without roots enhances the likelihood of dispersal in the form of seedlings to other locations. Seeds of many *Garcinia* species are dispersed by animals of various sizes [64,65,66]. Evidence is accumulating that removing primary roots at different stages of development does not affect germination or seedling growth in *Garcinia* species [12]. Our study did not support multiple root emergence in *G. aristata*; thus, having shoot emergence without roots could bait some animals, propelling dispersal. For seedlings to grow without the help of roots, storage tissues must supply reserves for growing seedlings. Whilst it is not known which tissue supplies nutrients to the growing seedling, we conjecture that the hypocotyl is somehow involved. Although more studies are required to test these hypotheses, we presume these explanations work inclusively.

## Figures and Tables

**Table 1:** Morphophysiological traits of fresh seeds of *Garcinia aristata*.

Seed Traits	Mean (±SD)	Maximum Value	Minimum Value	Coefficient of Variation
Length (mm)	17.20 (2.62)	21.17	12.30	15.23
Width (mm)	11.40 (1.90)	13.71	7.82	16.71
Depth (mm)	9.75 (1.69)	13.50	6.36	17.34
Seed shape	0.05 (0.01)	0.09	0.02	25.58
Fresh mass (g)	2.01 (0.68)	3.07	0.65	33.89
Dry mass (g)	0.69 (0.22)	1.08	0.18	32.15
Initial moisture content (%)	67.22 (3.38)	76.10	61.04	5.03
Seed embryo dry mass (g)	0.61 (0.20)	1.00	0.14	33.24
Seed coat dry mass (g)	0.07 (0.03)	0.18	0.04	30.96
Seed coat ratio	0.11(0.04)	0.21	0.07	36.77
Probability of desiccation sensitivity	0.82 (0.13)	0.93	0.32	16.23
Increase in seed mass after imbibition (%)	12.02 (2.21)	15.38	10.02	8.39

**Table 2:** Analysis of deviance of GLM for the influence of light, temperature, and their interaction on final germination percentage of fresh seeds of *Garcinia aristata*.

Factor	*df*	Deviance	Resid. Df	Resid. Dev	*p* (>*Chi*)
Null			39	481.48	
Light	1	0.05	38	481.43	0.8252
Temperature	3	466.71	35	14.72	0.0001
Light × Temperature	3	3.64	32	11.08	0.3035

## Data Availability

All the data used in this work are presented in the manuscript.

## References

[1] Fenner M., Thompson K. (2005). The Ecology of Seeds.

[2] Finch-Savage W.E., Leubner-Metzger G. (2006). Seed dormancy and the control of germination. New Phytol..

[3] Nonogaki H., Bassel G.W., Bewley J.D. (2010). Germination—Still a mystery. Plant Sci..

[4] Bewley J.D., Bradford K.J., Hilhorst H.W., Nonogaki H. (2013). Seeds: Physiology of Development and Germination.

[5] ISTA (2023). International Rules for Seed Testing.

[6] Baskin J.M., Baskin C.C. (2021). The great diversity in kinds of seed dormancy: A revision of the Nikolaeva–Baskin classification system for primary seed dormancy. Seed Sci. Res..

[7] Tutin T. (1938). The autecology of *Zostera marina* in relation to its wasting disease. New Phytol..

[8] Taylor A. (1957). Studies of the development of *Zostera marina* L.: II. Germination and seedling development. Can. J. Bot..

[9] Kuo J., Iizumi H., Nilsen B.E., Aioi K. (1990). Fruit anatomy, seed germination and seedling development in the Japanese seagrass Phyllospadix (Zosteraceae). Aquat. Bot..

[10] Zhu M., Zang Y., Zhang X., Shang S., Xue S., Chen J., Tang X. (2023). Insights into the regulation of energy metabolism during the seed-to-seedling transition in marine angiosperm *Zostera marina* L.: Integrated metabolomic and transcriptomic analysis. Front. Plant Sci..

[11] Park J.-I., Kim J.H., Song H.-J., Kim G.Y. (2022). Effects of Light, Salinity and Temperature on Germination Characteristics of Surfgrass, *Phyllospadix japonicus* and *P. iwatensis* Seeds. Ocean. Sci. J..

[12] Joshi G., Kumar A.A., Gowda B., Srinivasa Y. (2006). Production of supernumerary plants from seed fragments in *Garcinia gummi-gutta*: Evolutionary implications of mammalian frugivory. Curr. Sci..

[13] Goh H.-H., Bakar S.A., Azlan N.D.K., Zainal Z., Noor N.M. (2019). Transcriptional reprogramming during *Garcinia*-type recalcitrant seed germination of *Garcinia mangostana*. Sci. Hortic..

[14] Agyili J., Sacande M., Koffi E., Peprah T. (2007). Improving the collection and germination of West African *Garcinia kola* Heckel seeds. New For..

[15] Ha C., Sands V., Soepadmo E., Jong K. (1988). Reproductive patterns of selected understorey trees in the Malaysian rain forest: The sexual species. Bot. J. Linn. Soc..

[16] Vogel E.d. (1982). Seedlings of Dicotyledons.

[17] Baskaware S.V., Deodhar M.A. (2023). Apomixis and Sexual Systems in Various Species of *Garcinia* with Special Reference to *Garcinia Indica* (Thouars) Choisy. Int. J. Fruit Sci..

[18] Teo C.K. (1992). In vitro culture of the mangosteen seed. Acta Hortic..

[19] Cardoso A.A., Barbosa S., Santos B.R. (2021). Optimum conditions for seed propagation of *Garcinia brasiliensis*: Mimicking natural habitats allows better results. J. Seed Sci..

[20] Malik S., Chaudhury R., Abraham Z. (2005). Seed morphology and germination characteristics in three *Garcinia* species. Seed Sci. Technol..

[21] Corbineau F., Côme D. (1986). Experiments on the storage of seeds and seedlings of *Symphonia globulifera* Lf (Guttiferae). Seed Sci. Technol..

[22] Kanmegne G., Omokolo N.D. (2008). Germination of *Garcinia kola* (Heckel) seeds in response to different hormone treatments. Fruits.

[23] Normah M., Rosnah H., Noor-Azza A. (1992). Multiple shoots and callus formation from seeds of mangosteen (*Garcinia mangostana* L.) cultured in vitro. Acta Hortic..

[24] Osman M.B., Milan A.R. (2006). Mangosteen: Garcinia mangostana L..

[25] Roberts E. (1973). Predicting the storage life of seeds. Seed Sci. Technol..

[26] Malik S., Chaudhury R., Abraham Z. (2005). Desiccation-freezing sensitivity and longevity in seeds of *Garcinia indica*, *G. cambogia* and *G. xanthochymus*. Seed Sci. Technol..

[27] Normah M.N., Aizat W.M., Hussin K., Rohani E.R. (2016). Seed characteristics and germination properties of four *Garcinia* (Clusiaceae) fruit species. Fruits.

[28] SID (2023). SER, INSR, RBGK, Seed Information Database (SID). https://ser-sid.org/about.

[29] Joshi G., Phartyal S., Arunkumar A. (2017). Non-deep physiological dormancy, desiccation and low-temperature sensitivity in seeds of *Garcinia gummi-gutta* (Clusiaceae): A tropical evergreen recalcitrant species. Trop. Ecol..

[30] Mathew K., George S.T. (1995). Dormancy and storage of seeds in *Garcinia cambogia* Desr.(Kodampuli). J. Trop. Agric..

[31] Chacko K., Pillai P. (1997). Seed characteristics and germination of *Garcinia gummi-gutta* (L.) Robs. Indian For..

[32] Anilkumar C., Babu K., Krishnan P. (2002). Dormancy mechanism and effects of treatments on the germination of *Garcinia gummi-gutta* (Clusiaceae) seeds. J. Trop. Sci..

[33] Subbiah A., Ramdhani S., Pammenter N.W., Macdonald A.H. (2019). Towards understanding the incidence and evolutionary history of seed recalcitrance: An analytical review. Perspect. Plant Ecol. Evol. Syst..

[34] Jaganathan G.K. (2021). Ecological insights into the coexistence of dormancy and desiccation-sensitivity in Arecaceae species. Ann. Sci..

[35] Tweddle J.C., Dickie J.B., Baskin C.C., Baskin J.M. (2003). Ecological aspects of seed desiccation sensitivity. J. Ecol..

[36] Guedje N.M., Lejoly J., Nkongmeneck B.-A., Jonkers W.B. (2003). Population dynamics of *Garcinia lucida* (Clusiaceae) in Cameroonian Atlantic forests. For. Ecol. Manag..

[37] Liu Y., Qiu Y.P., Zhang L., Chen J. (2005). Dormancy breaking and storage behavior of *Garcinia cowa* Roxb. (Guttiferae) seeds: Implications for ecological function and germplasm conservation. J. Integr. Plant Biol..

[38] Ancy J., Satheeshan K., Jomy T. (2007). Seed germination studies in *Garcinia* spp.. J. Spices Aromat. Crops..

[39] Ng F. (1979). Germination ecology of Malaysian woody plants IV. Malays. For..

[40] Ng F. (1973). Germination of fresh seeds of Malaysian trees. Malays. For..

[41] Viana W.G., Lando A.P., Silva R.A.d., Costa C.D.d., Vieira P.H.M., Steiner N. (2020). Physiological performance of *Garcinia gardneriana* (Planch. & Triana) Zappi: A species with recalcitrant and dormant seeds. J. Seed Sci..

[42] Borhidi A. (1980). New names and new species in the Flora of Cuba, II. Acta Bot. Acad. Sci. Hung..

[43] Greuter W., Rankin R. (2022). Vascular Plants of Cuba. A Checklist. Third, Updated Edition of The Spermatophyta of Cuba.

[44] González-Oliva L., González-Torres L.R., Palmarola A., Barrios D., Testé E. (2015). Categorización de taxones de la flora de Cuba—2015. Bissea.

[45] Fuentes I.M., González-Oliva L., Baró I., González M.T., Mancina C.A. (2019). Efecto potencial del cambio climático sobre la distribución de plantas asociadas a bosques húmedos del oriente de Cuba. Acta Botánica Cuba.

[46] González-Torres L.R., Palmarola A., González-Oliva L., Bécquer E.R., Testé E., Barrios D. (2016). Lista Roja de la Flora de Cuba. Bissea.

[47] Sánchez J.A., Montejo L.A., Gamboa A., Albert-Puentes D., Hernández F. (2015). Germinación y dormancia de arbustos y trepadoras del bosque siempreverde de la Sierra del Rosario, Cuba. Pastos y Forrajes.

[48] Sánchez J.A., Pernús M., Martínez J. (2020). Tratamientos de semillas para mejorar la germinación de *Guazuma ulmifolia* bajo estrés hídrico y calórico: Comparación entre árboles tropicales pioneros. Rev. Jard. Bot. Nac. Univ. Habana.

[49] Borhidi A. (1996). Phytogeography and Vegetation Ecology of Cuba.

[50] Fick S.E., Hijmans R.J. (2017). WorldClim 2: New 1-km spatial resolution climate surfaces for global land areas. Int. J. Climatol..

[51] Thompson K., Band S.R., Hodgson J.G. (1993). Seed size and shape predict persistence in soil. Funct. Ecol..

[52] Daws M., Garwood N.C., Pritchard H. (2006). Prediction of desiccation sensitivity in seeds of woody species: A probabilistic model based on two seed traits and 104 species. Ann. Bot..

[53] Baskin J.M., Davis B.H., Baskin C.C., Gleason S.M., Cordell S. (2004). Physical dormancy in seeds of *Dodonaea viscosa* (Sapindales, Sapindaceae) from Hawaii. Seed Sci. Res..

[54] Villela F.A., Filho L.D., Sequeira E.L. (1991). Tabela de potential osmótico em função da concentração de polietileno glicol 6.000 e da temperatura. Pesqui. Agropecu. Bras..

[55] Michel B.E., Kaufmann M.R. (1973). The osmotic potential of polyethylene glycol 6000. Plant Physiol..

[56] Bolker B.M., Brooks M.E., Clark C.J., Geange S.W., Poulsen J.R., Stevens M.H.H., White J.S.S. (2009). Generalized linear mixed models: A practical guide for ecology and evolution. Trends Ecol. Evol..

[57] Horo S., Ranjani R., Waman A.A., Bohra S.K.J.a.P. (2022). Seed Germination Studies in Andaman Kokum (*Garcinia dhanikhariensis* SK Srivastava): An Endemic Species from Bay Islands, India. J. Andaman. Sci. Assoc..

[58] Asomaning J., Olympio N., Sacande M. (2011). Desiccation sensitivity and germination of recalcitrant *Garcinia kola* heckel seeds. Res. J. Seed Sci..

[59] Onyekwelu S. (1987). Germination and seedling morphology of *Garcinia kola* Heckel. J. Trop. For. Resour..

[60] Baskin C.C., Baskin J.M. (2014). Seeds: Ecology, Biogeography, and Evolution of Dormancy and Germination.

[61] Pritchard H.W., Tsan F.Y., Wen B., Jaganathan G.K., Calvi G., Pence V.C., Mattana E., Ferraz I.D., Seal C.E. (2022). Regeneration in Recalcitrant-Seeded Species and Risks from Climate Change (Plant Regeneration from Seeds).

[62] Nzegbule E., Mbakwe R. (2001). Effect of pre-sowing and incubation treatment on germination of *Garcinia kola* (Heckel) seeds. Fruits.

[63] Kranner I., Minibayeva F.V., Beckett R.P., Seal C.E. (2010). What is stress? Concepts, definitions and applications in seed science. New Phytol..

[64] Wang Z., Wang B., Yi X., Yan C., Zhang Z., Cao L. (2019). Re-caching behaviour of rodents improves seed dispersal effectiveness: Evidence from seedling establishment. For. Ecol. Manag..

[65] Anto M., Jothish P., Angala M., Anilkumar C. (2018). Fruit predation and adaptive strategies of *Garcinia imberti*, an endangered species of southern Western Ghats. Curr. Sci..

[66] McConkey K.R., Brockelman W.Y., Saralamba C., Nathalang A. (2015). Effectiveness of primate seed dispersers for an “oversized” fruit *Garcinia benthamii*. Ecology.

